# Regulation of H-Ras-driven MAPK signaling, transformation and tumorigenesis, but not PI3K signaling and tumor progression, by plasma membrane microdomains

**DOI:** 10.1038/oncsis.2016.36

**Published:** 2016-05-30

**Authors:** J V Michael, J G T Wurtzel, L E Goldfinger

**Affiliations:** 1 Department of Anatomy and Cell Biology, The Sol Sherry Thrombosis Research Center, Temple University School of Medicine, Philadelphia, PA, USA; 2 Cancer Biology Program, Fox Chase Cancer Center, Philadelphia, PA, USA

## Abstract

In this study, we assessed the contributions of plasma membrane (PM) microdomain targeting to the functions of H-Ras and R-Ras. These paralogs have identical effector-binding regions, but variant C-terminal targeting domains (tDs) which are responsible for lateral microdomain distribution: activated H-Ras targets to lipid ordered/disordered (L_o_/L_d_) domain borders, and R-Ras to L_o_ domains (rafts). We hypothesized that PM distribution regulates Ras-effector interactions and downstream signaling. We used tD swap mutants, and assessed effects on signal transduction, cell proliferation, transformation and tumorigenesis. R-Ras harboring the H-Ras tD (R-Ras-tH) interacted with Raf, and induced Raf and ERK phosphorylation similar to H-Ras. R-Ras-tH stimulated proliferation and transformation *in vitro*, and these effects were blocked by both MEK and PI3K inhibition. Conversely, the R-Ras tD suppressed H-Ras-mediated Raf activation and ERK phosphorylation, proliferation and transformation. Thus, Ras access to Raf at the PM is sufficient for MAPK activation and is a principal component of Ras mitogenesis and transformation. Fusion of the R-Ras extended N-terminal domain to H-Ras had no effect on proliferation, but inhibited transformation and tumor progression, indicating that the R-Ras N-terminus also contributes negative regulation to these Ras functions. PI3K activation was tD independent; however, H-Ras was a stronger activator of PI3K than R-Ras, with either tD. PI3K inhibition nearly ablated transformation by R-Ras-tH, H-Ras and H-Ras-tR, whereas MEK inhibition had a modest effect on Ras-tH-driven transformation but no effect on H-Ras-tR transformation. R-Ras-tH supported tumor initiation, but not tumor progression. While H-Ras-tR-induced transformation was reduced relative to H-Ras, tumor progression was robust and similar to H-Ras. H-Ras tumor growth was moderately suppressed by MEK inhibition, which had no effect on H-Ras-tR tumor growth. In contrast, PI3K inhibition markedly suppressed tumor growth by H-Ras and H-Ras-tR, indicating that sustained PI3K signaling is a critical pathway for H-Ras-driven tumor progression, independent of microdomains.

## Introduction

The Ras superfamily comprises over 150 small GTPases, which can bind and hydrolyze guanine triphosphate (GTP). The proto-oncogenic homologs most prominently associated with cancers, H-, N- and K-Ras, are ubiquitously expressed and have overlapping yet non-redundant functions.^1, 2, 3, 4^ Ras propagates growth factor signaling pathways regulating cell proliferation, differentiation, angiogenesis and survival.^1, 2, 3^ Ras proteins cycle between an active GTP-bound and an inactive GDP-bound state. Constitutively active (CA) (that is, GTP-locked) Ras mutations are highly transforming and induce tumor formation,^5^ and the combined set of activating Ras mutations altogether are associated with as high as ~30% of human malignancies.^6^ However, each isotype displays tissue type specificities in cancers, indicating context-dependent modes of action.^6^ Thus, the molecular mechanisms of isotypic Ras oncogenesis are still not completely understood.^1, 2, 3^

Related-Ras 1 (R-Ras) is a highly conserved Ras paralog with limited mitogenic signaling properties. R-Ras functions include cell motility, survival and vascular quiescence, distinct from other Ras paralogs.^7, 8, 9, 10, 11, 12^ CA R-Ras weakly transforms select cell lines, and activating mutations are not prominent in human malignancies.^13, 14^ However, despite identical effector-binding domains, H- and R-Ras support distinct effector signal outputs, suggesting contributions from other domains in the regulation of Ras-effector interactions and signaling. H- and R-Ras paralogs are most divergent in the termini: a 26 amino-acid N-terminal extension in R-Ras which is absent from H-Ras, and in the C-terminal hypervariable domains (HVR), which harbor membrane targeting domains (tDs) at the C-termini. The R-Ras N-terminal domain regulates R-Ras-dependent cell migration, but has not been associated with mitogenic signaling.^15^ Activated H-Ras interacts in cells with Raf kinase and facilitates its activation, which propagates phosphorylation of cytosolic kinases MEK and ERK, driving cell proliferation.^16, 17, 18^ R-Ras is capable of binding Raf *in vitro*; however this interaction either does not occur or is of low affinity in cells, believed to account for the weak transforming function of R-Ras.^19, 20^ The mechanism for these distinct behaviors is not understood but may reflect distinct properties based on the HVRs.

Ras proteins are initially synthesized as globular, cytoplasmic polypeptides, which undergo a series of irreversible lipid modifications within the C-terminal tDs that increase the hydrophobicity of the C-termini and support anchorage of the Ras proteins to ER membranes and subsequent transport to the Golgi, before delivery to the plasma membrane (PM).^21^ The K-Ras tD contains Lys-rich sequences, which enhance charge-based PM interactions.^22^ In contrast, the tDs in H-, N- and R-Ras also contain adjacent target sites for reversible palmitoylation by a thioester linkage (C181 and C184 in H-Ras, C213 in R-Ras).^23, 24^ Palmitoylation occurs at the Golgi,^25, 26^ and is required for sorting into post-Golgi vesicles and vesicular transport to the PM; depalmitoylation at the PM by cognate palmitoyl (acyl) thioesterases completes the cycle by driving retrograde recycling to the Golgi.^12, 27, 28, 29, 30, 31^ Ras isotypes also have distinct distributions in microdomains at the PM, driven by guanine nucleotide loading as well as palmitoylation.^30, 31, 32, 33^ H-Ras is anchored to the lipid ordered (L_o_, that is, lipid raft) membrane while GDP loaded, and is shuttled to the lipid ordered/lipid disordered (L_o_/L_d_) border upon GTP loading.^34, 35, 36^ R-Ras preferentially anchors within the L_o_ domain, regardless of activation state.^34, 37^ These membrane microdomain preferences are supported by electron microscopy and *in silico* simulations.^34, 36, 37^ Efficient activation of Raf by H-Ras requires signaling from the L_d_ membrane.^36, 38, 39^ We hypothesized that the lateral membrane distributions of H-Ras and R-Ras are key determinants of their distinct mitogenic and oncogenic properties. In this study, we investigated the spatial regulation of Ras/Raf interaction and signal propagation by PM microdomain Ras targeting, and the contributions of microdomain-dependent signaling to Ras-induced cell proliferation and tumorigenesis.

## Results

### The H-Ras tD is both necessary and sufficient for palmitoylated Ras/Raf interaction, Raf-1 activation and MAPK signal propagation in cells

**Figure 1:**
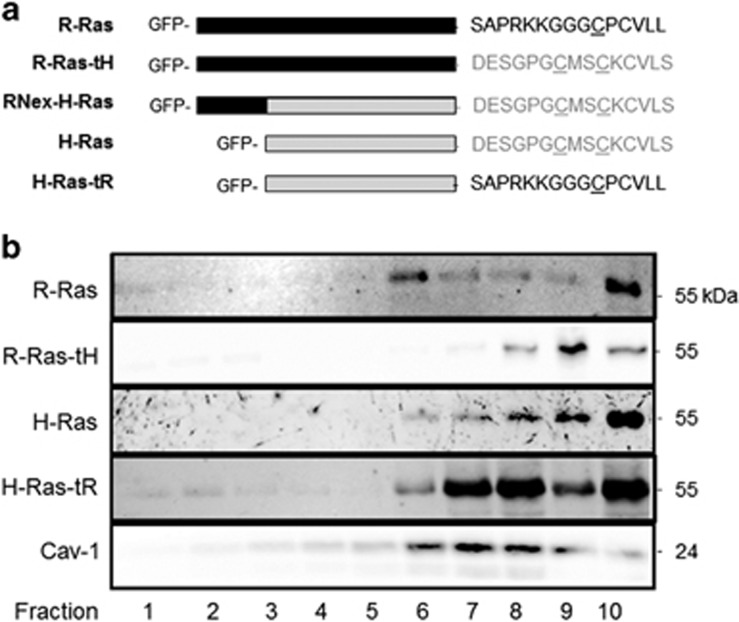
Ras targeting domain swap mutants determine PM microdomain localization. (**a**) Schematic representation of GFP-Ras expression constructs used in these studies. R-Ras-tH, R-Ras(1–203)H-Ras(175–189); H-Ras-tR, H-Ras(1–174)-R-Ras(204–218); RNex-H-Ras, R-Ras(1–26)-H-Ras(1–189). Black bar, R-Ras G domain; gray bar, H-Ras G domain; underline, palmitoylation sites; lower case, geranylgeranylation site; *, farnesylation site. (**b**) NIH3T3 cells stably expressing the indicated GFP-Ras variants were fractionated by ultracentrifugation using a step-gradient from 10 to 45% sucrose followed by immunoblotting with α-GFP antibodies. α-Cav-1 was used as a lipid raft marker. All blots representative of three independent experiments.

We created tD switch mutants between H- and R-Ras on the CA background (H-Ras(G12V), R-Ras(G38V)), in which the final 15 amino acids were swapped (Figure 1a), as GFP fusions at the Ras N-terminus. We confirmed the tD-dependent membrane microdomain distributions of these variants by sucrose fractionation.^40^ As predicted, active H-Ras was only partially enriched in Cav-1-positive (L_o_ domain) fractions, while highly enriched in dense Cav-1-negative fractions. R-Ras was enriched in Cav-1-positive fractions, confirming that activated H-Ras targets to the L_o_/L_d_ border whereas activated R-Ras is primarily sequestered in the L_o_ domain. H-Ras harboring the R-Ras tD (hereafter referred to as H-Ras-tR) was highly enriched in Cav-1-positive fractions as observed previously,^37^ whereas R-Ras harboring the H-Ras tD (R-Ras-tH) was minimally in Cav-1 fractions, and was highly enriched in the dense L_d_ fractions (Figure 1b). Thus, the H-Ras targeting domain (tH) and the R-Ras targeting domain (tR) enforced isotypic lateral targeting of Ras proteins to the L_o_/L_d_ border or L_o_ domain, respectively.

**Figure 2:**
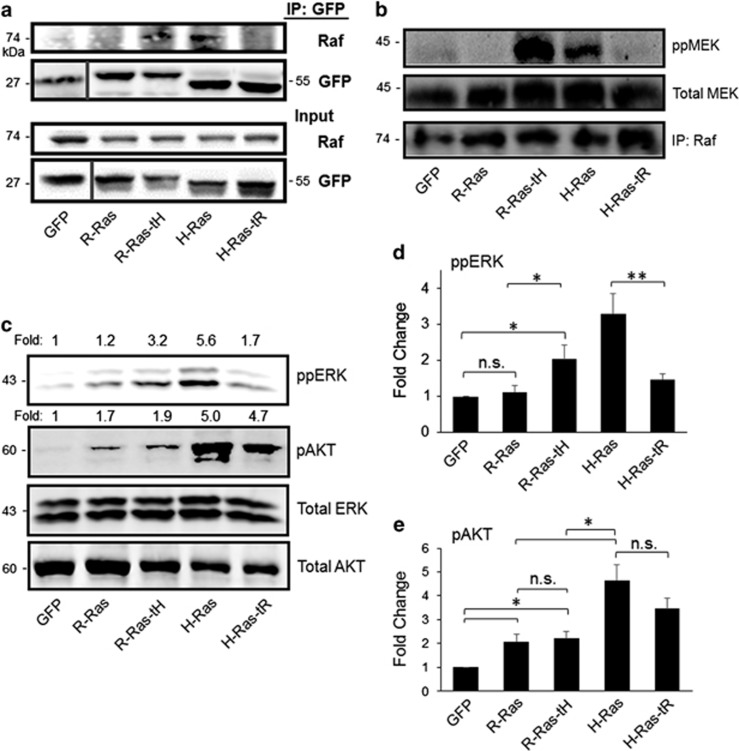
Ras targeting domains dictate access to Raf and MAPK signaling. (**a**) NIH3T3 murine fibroblasts were stably transfected with GFP-tagged Ras variants as indicated, and GFP fusion proteins were immunoprecipitated (IP) from cell lysates (Input) with α-GFP antibodies, followed by immunoblotting with α-Raf or α-GFP antibodies. (**b**) Raf kinase assay. Cells were serum-starved, and Raf activity was assessed as described in Materials and methods. Immunoblotting of the IP fraction with α-Raf antibodies is shown in the lower panel. (**c**) ERK and AKT activation in Ras-expressing cells after 72 h serum starvation, as assessed by immunoblotting of cell lysates with the indicated antibodies. Phospho:total ratios are shown above the respective blots as a ratio to GFP. (**d**, **e**) Fold change in phospho-ERK:total ERK or phospho-AKT:total AKT ratios compared with GFP control+s.e.m. *P<0.05; **P<0.003, n.s., not significant. All blots representative of five independent experiments.

R-Ras can bind Raf-1 *in vitro*; however, R-Ras/Raf-1 interaction and downstream signaling has not been observed in cells.^19, 20^ To test whether membrane microdomain localization was responsible for this property of R-Ras, we investigated the effect of mistargeted H- and R-Ras on Raf interaction in cells. NIH3T3 cells stably-expressing GFP-Ras variants at similar protein levels (Figure 2a) were lysed and subjected to immunoprecipitation (IP) with GFP antibodies. Endogenous Raf-1 co-precipitated with H-Ras but was absent from R-Ras IP fractions, confirming that H-Ras but not R-Ras interacts with Raf-1 in these cells.^19, 41^ In contrast to R-Ras, Raf-1 was enriched in GFP-R-Ras-tH precipitate fractions. Conversely, the interaction of H-Ras with Raf-1 was abrogated with H-Ras harboring the tR (Figure 2a). Thus, PM microdomain localization is a critical determinant in Ras/Raf-1 interaction in cells, and the tH supports Ras/Raf-1 interaction. To address whether interaction of Ras with Raf correlated with Raf-1 activation, we assessed Raf-1 kinase activity in serum-deprived cells expressing Ras variants. Endogenous Raf-1 isolated (by IP) from cells expressing H-Ras and R-Ras-tH, but not R-Ras or H-Ras-tR, was capable of phosphorylating recombinant MEK *in vitro* (Figure 2b). Thus, the tH is both necessary and sufficient for Ras-mediated Raf-1 recruitment to the membrane and interaction of a Ras protein with Raf-1 in cells, and this interaction facilitates Raf-1 activation.

To investigate downstream signaling of the tD mutants, we assessed phosphorylation of ERK and AKT (ppERK (T202/Y204) and pAKT (S473), indicating MEK and PI3K activation, respectively) in serum-starved cells expressing Ras-tD variants. R-Ras did not stimulate ERK phosphorylation, consistent with previous reports.^19^ Conversely, H-Ras as well as R-Ras-tH stimulated phosphorylation of ERK in serum-starved cells. H-Ras-tR yielded a marked reduction in ppERK compared with H-Ras, although these phosphorylation events were not completely blocked (Figures 2c and d). Thus, H-Ras stimulation of ERK phosphorylation is tD dependent. We observed similar results in HEK293 cells transiently transfected with GFP or the GFP-Ras variants, indicating that Ras-tD-dependent MAPK signaling is not cell-type specific (Supplementary Figures 1a and b). Moreover, R-Ras, R-Ras-tH, H-Ras and H-Ras-tR each promoted robust AKT phosphorylation, regardless of the tD, but H-Ras was a more potent activator of PI3K than R-Ras (Figures 2c and e; Supplementary Figures 1a and c). Thus, the Ras-tH domain regulates Ras access to Raf-1 in cells, and facilitates activation of the MAPK pathway. In contrast, both H-Ras and R-Ras can activate PI3K independent of microdomain localization.

### The H-Ras tD is necessary for Ras-induced cell proliferation

**Figure 3:**
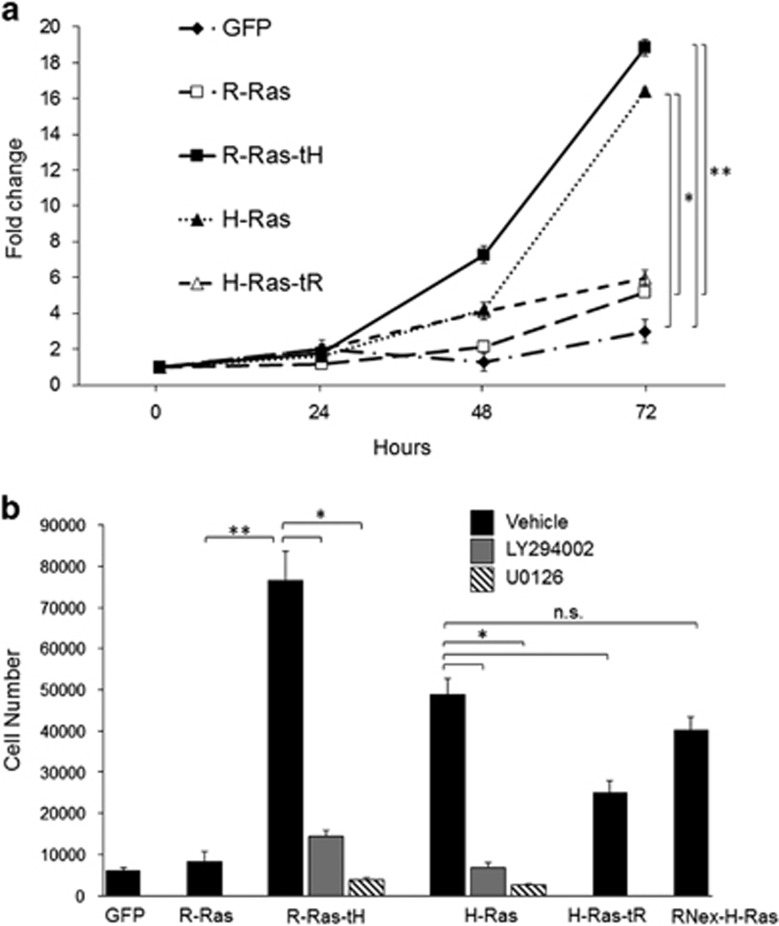
The tH is necessary for a Ras protein to promote cell proliferation. 1 × 10^4^ NIH3T3 cells stably expressing the indicated Ras mutants were seeded in triplicate wells in media with low serum. (**a**) Cells were harvested and counted at the indicated intervals. Cells were harvested and counted 5 h after seeding for 0-h time points. (**b**) Cells were plated in the presence of either 20 μm LY294002 or 30 μm U0126 as indicated and counted at 72 h. *P<0.01; **P<0.002, n.s., not significant. Representative of five independent experiments.

We investigated the effect of Ras targeting on cell proliferation. Cells stably expressing the Ras chimeras were maintained in low serum conditions, and cell growth was assessed over 72 h. These conditions resulted in inhibited cell growth and maintenance in G_0_/G_1_ in GFP- and R-Ras-expressing cells, as well as H-Ras-tR cells, as evidenced by DNA labeling and FACS, whereas H-Ras and R-Ras-tH cells showed population shifts toward S/G2, suggesting induction of mitogenesis in these cells. Each population had few dead cells, indicating that the Ras variants combined with low serum growth conditions did not result in substantial cell death (Supplementary Figure 2). To assess the ability of the Ras variants to promote cell proliferation, Ras-expressing cells were maintained in low serum conditions and cell population counts were monitored over time. Cell growth was significantly retarded by low serum conditions in control cells expressing vector (GFP) alone, whereas both H-Ras- and R-Ras-tH-expressing cells proliferated rapidly under these conditions. R-Ras and H-Ras-tR expression had little effect on stimulating cell growth over control (Figure 3a). To investigate whether these proliferation effects were cell-type specific, we monitored proliferation of HEK293 cells in low serum, after transient transfection with Ras variants. These cells showed similar trends in proliferation as the stably-expressing Ras NIH3T3 cells: R-Ras-tH and H-Ras potently stimulated proliferation, whereas R-Ras and H-Ras-tR did not (Supplementary Figure 3). Thus, H-Ras-typic targeting supports Ras-induced cell proliferation, while R-Ras-typic targeting does not support proliferation.

Next, we addressed effector pathways important for Ras-tH-induced cell growth. Cultured cells expressing the Ras chimeras were placed in low serum conditions in the presence of either 30 μm U0126 or 20 μm LY294002 (MEK and PI3K inhibitors, respectively). We confirmed that these inhibitors blocked their respective targets in these cells: LY294002 inhibited pAKT but not ERK, and conversely, U0126 inhibited ppERK but not pAKT, in serum-starved Ras cells (Supplementary Figure 4). Although R-Ras-tH strongly promoted proliferation, we observed a marked reduction in R-Ras-tH-driven cell growth with either LY294002 or U0126 treatment (Figure 3b). Similarly, H-Ras-induced proliferation was drastically reduced by blockade of either PI3K or MEK, indicating that both pathways are critical for Ras-induced cell proliferation.

In addition to the tDs, R-Ras and H-Ras are primarily distinguished by a non-conserved 26 amino-acid N-terminal extended domain in R-Ras (‘RNex'). To address a potential contribution of this R-Ras domain to Ras-induced cell growth, we stably expressed a GFP-Ras fusion harboring the RNex domain fused at the N-terminus to H-Ras(G12V) (hereafter referred to as RNex-H-Ras) (Figure 1a). RNex-H-Ras stable transfectants proliferated at a rate similar to H-Ras cells under low serum conditions, indicating that the RNex domain is not a major contributor to Ras-induced cell growth (Figure 3b). Thus, the Ras-tD is the critical domain dictating Ras-induced cell growth, which requires both PI3K and MAPK pathway activation by Ras.

### The H-Ras tD modulates Ras-induced cell transformation

**Figure 4:**
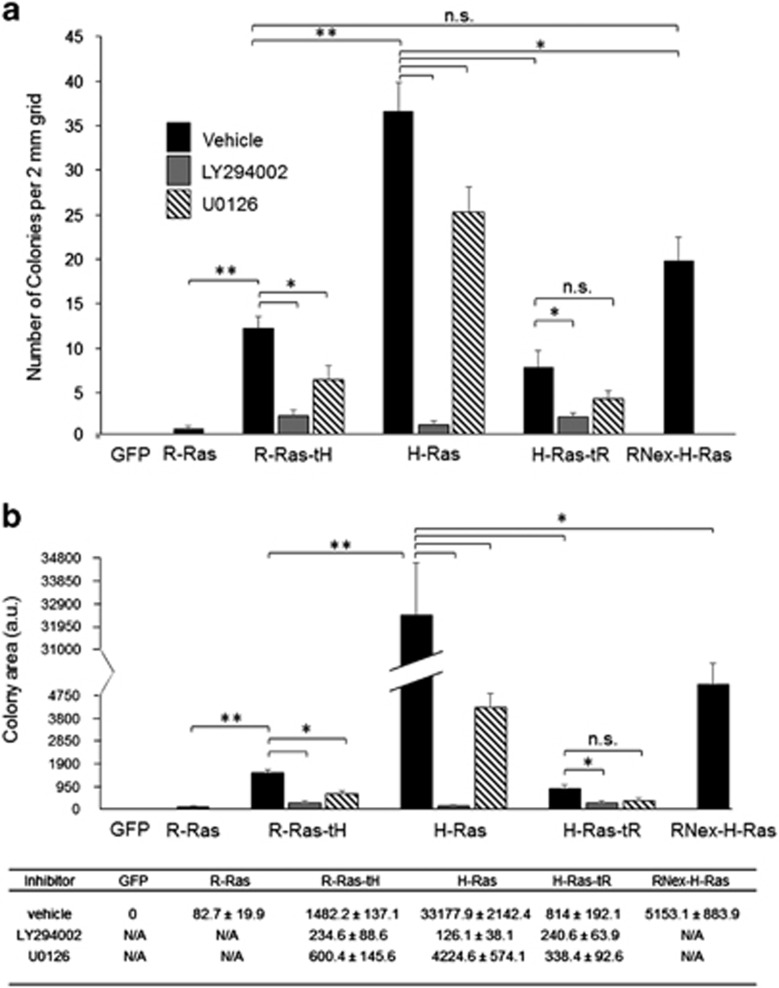
The H-Ras tD modulates Ras-induced cell transformation. 5 × 10^4^ cells were seeded into 0.3% agar for 14 days, and cells were fixed and imaged. (**a**) Number of colonies per 2 mm grid are shown. (**b**) Area of colonies per 2 mm grid were measured using ImageJ software. Average total areas per grid are shown+s.e.m. *P<0.001; **P<2 × 10^−6^. n.s., not significant. *n*=6. The values for the histogram in (**b**) are shown below in tabular form, ±s.e.m.

To investigate the contributions of Ras targeting to cellular transformation, we evaluated anchorage-independent growth by assessing colony formation of Ras stably transfected cells in soft agar. R-Ras stable transfectants produced minimal colony formation, similar to the baseline control. H-Ras supported formation of many large colonies, whereas H-Ras-tR expression yielded significantly attenuated colony formation (Figures 4a and b). Thus, sequestration of H-Ras in the L_o_ domain attenuated but did not ablate its ability to promote cellular transformation. R-Ras-tH stimulated colony formation, indicating a gain of function for R-Ras by fusion of the tH domain (Figure 4a). However, although R-Ras-tH expression yielded an increase in colony formation compared with parental R-Ras, with both the number and size of the colonies greater than those produced by R-Ras cells, the R-Ras-tH colonies were fewer in number and smaller than those formed by H-Ras (Figures 4a and b). Colony formation by RNex-H-Ras-expressing cells was similar to that in R-Ras-tH cells, indicating that the RNex domain negatively regulates tH-targeted Ras-induced colony formation (Figures 4a and b).

MEK inhibition partially inhibited colony formation in H-Ras and Ras-tH cells, but had no effect on the already diminished colony growth by H-Ras-tR cells. In contrast, PI3K inhibition ablated Ras-induced colony formation by H-Ras, R-Ras-tH and H-Ras-tR cells (Figure 4). Together, these data demonstrate that L_o_/L_d_ targeting by the tH domain facilitates Ras-induced cellular transformation through both PI3K and MEK signaling, and sustained PI3K signaling is a major determinant for Ras-driven transformation.

### Roles of the tDs in Ras-mediated tumorigenesis and tumor progression

**Figure 5:**
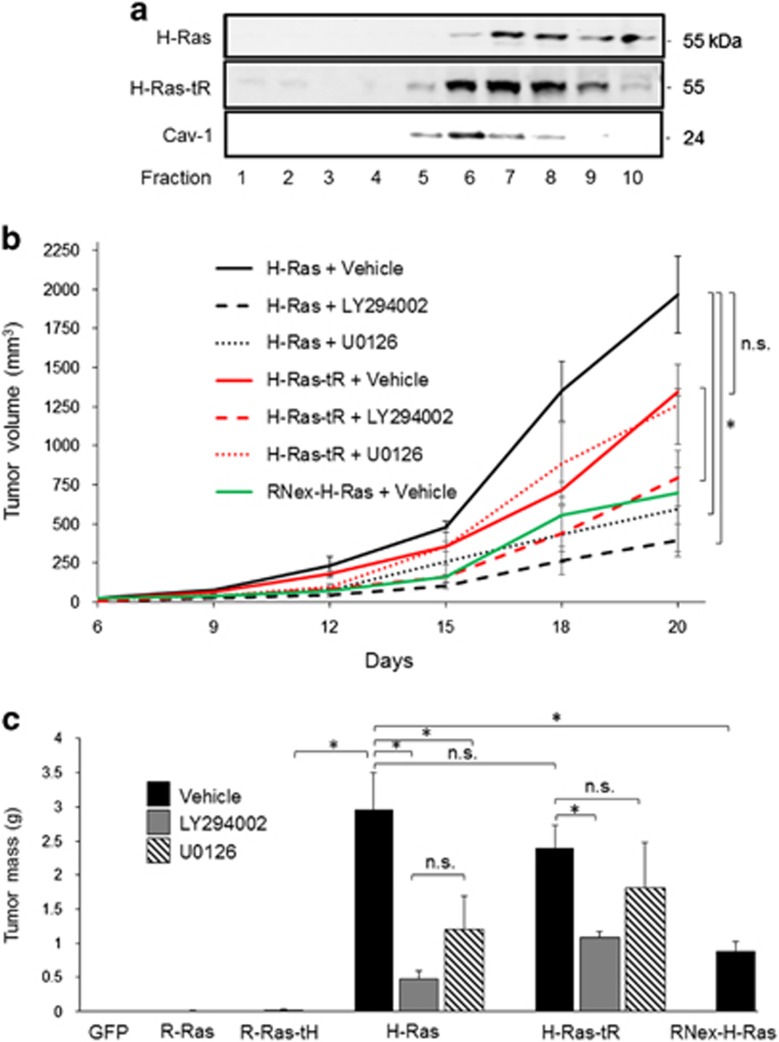
H-Ras is tumorigenic even with the R-Ras tD. (**a**) Cells stably expressing the indicated Ras variants were injected as a bolus allograft into the flanks of athymic mice. After 20 days, tumors were excised, and tumor lysates were fractionated by ultracentrifugation using a step-gradient from 10 to 45% sucrose. α-Cav-1 was used as a lipid raft marker. (**b**) Upon detection, tumor dimensions were measured every 72 h for volume calculation. (**c**) Average tumor masses of resected tumors at 20 days are shown+s.e.m. *P<0.05, n.s., not significant. *n*=10.

The distinct effects of Ras lateral membrane distribution on MAPK signaling, cell proliferation and transformation *in vitro* suggested a critical role of Ras microdomain localization at the PM in Ras-induced tumorigenesis. To address this possibility, we employed a tumor allograft model in which athymic mice were injected subcutaneously in each flank with Ras stable cells, and resultant tumors were resected after 20 days.^42^ We assessed membrane microdomain distribution of H-Ras variants in tumor lysates *ex vivo*, and found that the tR shifted H-Ras to the L_o_ domain, indicating that the predicted membrane microdomain distributions of these Ras variants were upheld in the tumor cells *in vivo* (Figure 5a). Cells expressing GFP alone produced no detectable tumors in any mice (Figure 5b, Table 1). R-Ras-expressing cells produced few tumors (that is, tumors only formed at 2 out of 13 allograft sites), and tumors that did form were <2 mm in diameter and appeared avascular, consistent with previous observations with R-Ras (Figure 5b, Table 1).^13, 14, 43, 44^ In contrast, R-Ras-tH cells produced tumors at 50% of injection sites, and average tumor mass in these cases was ~2.3-fold higher than those generated by cells expressing R-Ras, indicating that fusion of the tH domain enhances tumorigenesis by R-Ras. However, tumors produced by R-Ras-tH cells were small—one-tenth the size of tumors produced by H-Ras cells (Table 1). H-Ras cells formed large tumors at every injection site. RNex-H-Ras cell tumors grew at a slower rate than H-Ras cell tumors (Figures 5a and b), reflecting diminished colony formation in soft agar by the RNex fusion (Figure 4), and indicating that the RNex domain negatively regulates both H-Ras-driven transformation and tumor growth. Interestingly, H-Ras-tR cell lines formed tumors at every injection site, all of which were significantly larger than those generated by either of the R-Ras counterparts, and which more closely resembled H-Ras tumors (Figure 5b, Table 1). This was a surprising result, as we anticipated that tumor promotion by H-Ras-tR would be attenuated compared with H-Ras. Thus, Ras targeting is a critical factor for Raf-1 interaction, activation, MAPK signaling and cell transformation *in vitro* as well as tumor initiation *in vivo*; however, another determinant outside the Ras targeting domain is necessary for Ras-induced *in vivo* tumor progression.

To investigate signaling pathways important for Ras-driven tumorigenesis, either U0126 or LY294002 was administered intraperitoneally every 72 h starting at the time of seeding Ras cells, and tumor growth was monitored using calipers. Inhibition of MEK by U0126 administration resulted in ~70% attenuation of tumor growth by H-Ras cells, but had no effect on tumor growth by H-Ras-tR cells. However, blockade of PI3K by administration of LY294002 substantially inhibited growth of both H-Ras and H-Ras-tR tumors (Figures 5b and c). Thus, MAPK and PI3K signaling contributes to H-Ras-driven transformation and tumorigenesis, whereas PI3K activation is the major pathway driving tumor progression by lipid raft-sequestered H-Ras, which is deficient in MAPK signaling.

The effects of the tD swaps on Ras-induced signaling, proliferation, transformation and tumor progression are summarized in Table 2. Taken together, these data indicate that Ras membrane microdomain distribution regulates Raf pathway signaling and Ras-induced cell proliferation, and both MAPK and PI3K pathways are important for Ras-induced proliferation and transformation. However, Ras-induced PI3K signaling is tD independent, and this pathway is required to mediate H-Ras-induced tumor progression.

## Discussion

The results of this study demonstrate key roles of membrane microdomain targeting of Ras in signal transduction and stimulation of cell proliferation, transformation and tumor progression. H-Ras-typic targeting to the L_o_/L_d_ border at the PM was sufficient to support R-Ras/Raf interaction, and to facilitate activation of Raf-1 and ERK, providing a molecular mechanism for distinct MAPK signaling by H- and R-Ras. Moreover, Ras-induced proliferation was tD dependent, indicating that Ras signaling from the L_o_/L_d_ border is a critical determinant of Ras mitogenesis, and this function required both MAPK and PI3K pathway activation. However, PI3K activation by H-Ras was tD independent, and H-Ras with repressed MAPK activation by L_o_ sequestration was competent to promote tumor growth comparable to H-Ras, in a PI3K-dependent manner. Together, these studies demonstrate that the tD of H-Ras supports the ability of a Ras protein to drive MAPK signaling and cell proliferation, while PI3K activation is Ras-tD independent and is a major determinant of Ras-induced tumor progression.

Microdomain targeting of H-Ras and R-Ras was the critical determinant for Ras/Raf interaction. Early reports established that R-Ras was capable of binding Raf-1 *in vitro*,^20^ though later studies indicated that this interaction does not occur *in vivo*,^19^ alluding to spatial segregation of R-Ras and Raf-1 at the PM. Indeed, mistargeting R-Ras with swapped regions from the H-Ras HVR abrogates many of R-Ras' unique biological responses, including modulation of integrin activation, focal adhesion targeting and reactive oxygen species production, indicating a dominant role of the HVRs for functional distinctions between these proteins.^37, 38, 39, 45, 46^ In the current study, H-Ras sequestered within rafts via the tR domain did not robustly induce Raf-1 kinase activity or ERK phosphorylation, consistent with previous observations.^36, 39^ The marked reduction but incomplete ablation of H-Ras-induced MAPK signaling by the tR may reflect a scenario in which Ras-tR is enriched in L_o_ domains, but is not completely sequestered from the L_o_/L_d_ border where Raf can be accessed, which may account for conflicting reports on whether R-Ras supports minimal ERK activation, or none.^19, 47, 48^ This notion is supported by sucrose gradient fractionation data, which show that a small portion of tR-targeted Ras (R-Ras or H-Ras-tR) localized to the L_o_/L_d_ border or L_d_ domain. In an earlier study, H-Ras harboring an N-terminal raft-targeting domain from Lck was shown to activate ERK comparable to H-Ras(V12) in NIH3T3 cells.^49^ This discrepancy may reflect distinct orientation of the Ras-effector binding loop by membrane association with an N-terminal, as opposed to a C-terminal tag, which could alter effector interactions.^50, 51^ Thus, microdomain distribution appears to be the major determinant of Raf interaction and activation by H-Ras, and is sufficient for a Ras protein to propagate MAPK signaling.

Distinct from MAPK signaling, PI3K activation by H-Ras was tD independent, and these distinctions correlated with different pathway dependencies for H-Ras-induced proliferation, transformation and tumor progression. Ras microdomain-dependent Raf interaction and MAPK signaling corresponded to Ras mitogenesis, consistent with a requirement for Ras in Raf membrane recruitment and MEK/ERK and its roles in proliferation.^52, 53, 54, 55, 56^ However, PI3K pathway activation was also required for Ras-induced mitogenesis, indicating that both effector pathways are important for Ras stimulation of proliferation. Unlike cell proliferation, however, transformation by Ras reflected a much stronger dependence on PI3K signaling rather than MAPK signaling. The RNex domain, which is not conserved and is unique to R-Ras, negatively regulated H-Ras-driven transformation and tumor progression, demonstrating that microdomain localization is not the only factor separating H- and R-Ras functions. In a recent study, mice harboring Ras-binding mutations in the p110α subunit of PI3K showed diminished H-Ras-induced AKT phosphorylation and transformation of cells *ex vivo* despite no reduction in ERK phosphorylation, supporting the notion that PI3K signaling is a major pathway for Ras-induced transformation, consistent with our results.^57, 58^ These mice also showed defective K-Ras-driven tumorigenesis, which may reflect K-Ras-specific mechanisms of tumor promotion.^58^ Similarly, depletion of RalA, a major effector of RalGDS, diminished H-Ras-induced transformation and inhibited tumorigenesis, although RalA depletion had no effect on cell growth *in vitro,*^59^ underscoring that Ras-induced transformation and tumor promotion rely on distinct pathways from Ras mitogenesis. Moreover, we found that weak MAPK signaling was sufficient to support tumor initiation (R-Ras-tH), but not tumor progression. Ras-induced tumor progression was supported by MAPK-deficient, PI3K-competent H-Ras-tR, but not by R-Ras-tH, which showed weak activation of PI3K, and Ras tumor progression was blocked in all cases by inhibition of PI3K. Together, our data indicate that pathway activation driving Ras-induced cell proliferation does not correspond directly to Ras-mediated tumor progression, which specifically required strong PI3K pathway activation by Ras. Thus, while PM microdomain localization of Ras is critical for Ras-driven MAPK signaling and proliferation, and contributes to transformation, PI3K activation is Ras microdomain independent and is a key pathway in Ras-induced tumor progression.

Misregulated PI3K signaling occurs frequently in human cancers. Overstimulation of PI3K signaling may promote resistance to clinical therapies, which has sparked interest to improve current therapies or find new drug targets.^60, 61, 62, 63^ Notably, EGFR mutations, which are also common in human cancers, can stimulate PI3K signaling directly by Ras-independent pathways,^64, 65, 66^ which adds to the importance of misregulated PI3K signaling in oncogenesis. Our data support the notion that PI3K activation is a dominant pathway of Ras tumor progression, and is therefore an attractive target for increased efficacy in therapeutic intervention of H-Ras- and EGFR-mutant cancers.

## Materials and methods

### Antibodies, reagents and cDNAs

GFP (sc-9996), Raf (sc-133, sc-227) antibodies and purified recombinant MEK-1 (sc-4025) were from Santa Cruz Biotechnology (Santa Cruz, CA, USA). AKT (AH01112) and Phospho-AKT (44623G) were from Invitrogen (Waltham, MA, USA). p42/44 MAPK (ERK1/2), pp44/42 MAPK (ERK T202/Y204), ppMEK (S217/221) and pAKT (S473) antibodies were from Cell Signaling Technology (Danvers, MA, USA). Fluorophore-conjugated secondary antibodies were from LI-COR Biosciences (Lincoln, NE, USA). LY294002 was purchased from LC laboratories (Woburn, MA, USA). U0126 was purchased from Alfa Aesar (Ward Hill, MA, USA). Restriction endonucleases were obtained from New England Biolabs (Ipswich, MA, USA). pEGFP-C1 was from Clontech Laboratories (Mountain View, CA, USA). GFP-R-Ras constructs were made as described in Wurtzel *et al.*^12^ GFP-H-Ras G12V was a gift from K Svoboda (Addgene plasmid 18666). GFP-H-Ras (1–174)G12V- R-Ras (204–218) was generated from GFP-H-Ras G12V by PCR. GFP-RNex-H-Ras was generated by insertion of the first 26 amino acids of R-Ras from an R-Ras N-terminal domain construct originally described in Silver *et al.*,^67^ into the GFP-H-Ras background.

### Cell culture and transfection

NIH3T3 and HEK293 cells were obtained from American Type Culture Collection (Manassas, VA, USA) and tested for mycoplasma in our laboratory before use. NIH3T3 cells were maintained in Dulbecco's modified Eagle's medium (DMEM, Cellgro, Manassas, VA, USA) supplemented with 10% Bovine Calf Serum (BCS), 4 mm l-glutamine, 4500 mg/ml glucose, 50 U/ml penicillin, 50 μg/ml streptomycin sulfate and 1% non-essential amino acids (Sigma-Aldrich, St Louis, MO, USA) at 37 °C in 5% CO_2_. HEK293 cells were maintained in DMEM supplemented with 10% Fetal Bovine Serum (FBS). Cells were analyzed 24–48 h after transfection. For stable transfectants, transfected NIH3T3 cells were selected in media containing 2 μg/ml G418 Sulfate (Geneticin). Colonies were subcloned after 2 weeks in the continued presence of Geneticin with cloning cylinders, and transgene expression was confirmed by western blot analysis. Stable transfectants were maintained in 200 ng/ml Geneticin.

### Protein precipitation with perchloric acid

Cells lysates were collected and Bradford protein assays (Thermo Scientific, Waltham, MA, USA) were used to determine protein concentration. Total protein was precipitated with 6.6 m perchloric acid added to each sample for a final concentration of 0.66 m. Samples were incubated for 20 min at −20 °C and centrifuged at max r.p.m. for 15 min. Pellets were rinsed with water and centrifuged again, and resuspended in SDS buffer for SDS–PAGE.

### Immunoprecipitations and western blotting

Cells were cultured in complete DMEM and, in some cases, serum-starved by culturing in DMEM/0.2% serum. Cells were rinsed 2 × in PBS, and cell lysates were harvested by scraping in lysis buffer (10 mm Tris-Cl, pH 7.5, 100 mm NaCl, 2 mm MgOAc, 0.5% Nonidet P-40, 10 μm GTP, 1 mm Na_3_VO_4_, 20 μm β-glycerophosphate, 1 mm NaF, plus a cocktail of protease inhibitors; Roche, Madison, WI, USA). Insoluble material was removed by centrifugation. Fractions of the lysates were separated by SDS–PAGE, followed by western blotting (immunoblotting) with specific primary antibodies, followed by detection with infrared fluoriphore-conjugated secondary antibodies using fluorescence laser scanning (LI-COR Biosciences, Lincoln, NE, USA). For IPs, supernatants were pre-cleared by incubation with Protein G-coupled sepharose beads (Roche) for 1 h at 4 °C. Cleared lysates were incubated with 2 μg of antibody suspensions for 16 h at 4 °C, followed by antibody capture on protein G-sepharose beads for 1 h. Antibody-bound complexes were precipitated by centrifugation, washed and separated by SDS–PAGE, followed by western blotting with relevant antibodies.

### Raf kinase assay

NIH3T3 transfectants were maintained in serum-starved conditions and lysed as described above, followed by Raf-1 IP with specific antibodies. G-sepharose beads were washed 2 × with kinase buffer (20 mm MOPS, pH 7.2, 25 mm β-glycerophosphate, 5 mm EGTA, 1 mm Na_3_VO_4_, 1 mm dithiothreitol) before addition of kinase buffer containing 75 mm MgCl_2_, 0.5 mm ATP, and 0.4 μg recombinant MEK. Samples were incubated at 30°C on a rocker. Kinase reaction was stopped at 30 min by being boiled in SDS sample buffer.

### Cell proliferation

Cells were seeded at 1 × 10^4^ cells per well in 1% serum. Cells were collected by trypsinization and counted at 5 h for the initial time point, and represented as a ratio of total/initial cells for all other indicated time points. In some cases, 20 μm LY294002 or 30 μm U0126 was added to the culture medium. Cells were harvested and counted at the indicated times.

### Sucrose gradient cell fractionation

NIH3T3 cells were scraped and washed with PBS (with protease inhibitors), resuspended and lysed in 0.5 m Na_2_CO_3_ (pH 11.0) as described previously.^36^ Briefly, cells were passed through a 23-gauge needle 15 times, sonicated and centrifuged at 39 000 r.p.m. in a SW41TI rotor in a 10–45% (w/v) step sucrose gradient. Ten 950-μl fractions were collected from the top sucrose layer, and equivalent total protein fractions were separated by SDS–PAGE and examined by western blotting. Tumor lysates were Dounce homogenized in buffer containing protease inhibitors, passed 10 times through an 18-G needle, and processed as described above.

### Cell transformation

In all, 5 × 10^4^ cells were mixed with Noble agar such that the final agar concentration was 0.3%, supplemented with 10% FBS. This mixture was loaded into a 60-mm dish containing 0.6% Noble agar. Cells were fed every 4 days with 100 μl of medium containing 10% calf serum. In some cases, either 20 μm LY294002 or 30 μm U0126 was added to both the top and bottom layer of agar, and media was replaced every 4 days with media containing 10% FBS with the same concentration of inhibitor. After 14 days, cells were fixed with 10% MeOH/10% acetic acid for 10 min. Plates were stained with 0.01% crystal violet for 1 h, and colonies were visualized after light washes.

### Tumor growth in nude mice

Female NU(NCR)-Foxn1^nu^ mice (Charles River, Wilmington, MA, USA) at 6–7 weeks of age were divided into five groups of two animals each. Cells for allografts were harvested and suspended in Hanks balanced salt solution (Thermo Scientific) at a density of 5 × 10^6^/ml, and 200 μl of the cell suspension was injected subcutaneously into each flank. In some cases, 75 mg/kg LY294002, 30 mg/kg U0126, or vehicle only was administered every 72 h. Vehicle mice were given 200 μl of 5% DMSO. Tumor dimensions were measured using calipers, and tumor volume was calculated using the formula Volume=long axis × short axis^2^ × 0.52.^68^ Mice were killed at 20 days post injection, and resected tumors were fixed in 4% paraformaldehyde, and stored in PBS with 0.02% sodium azide. All animal experiments followed protocols approved by the Institutional Animal Care and Use Committee (IACUC) at Temple University, which requires compliance with NIH ethical regulations.

### Statistical analysis

One-way ANOVA followed by Fisher protected least significant difference analysis was used, using StatView (SAS Institute, Cary, NC, USA). A 5% probability was considered as significant. For *in vivo* studies, to detect a 30% difference between four groups using ANOVA with Bonferroni *post hoc* test (four tests), a minimum of five animals in each group would be needed, based on anticipated standard deviation=0.35, power=0.8 for *P*<0.05. The following web page was used for power calculation: http://www.stat.uiowa.edu/~rlenth/Power/index.html. Exclusion criteria included animal morbidity or premature death (none were observed). Tumor measurements were carried out by observers blinded to the allograft genotypes. Results are representative of three independent experiments with at least three replicates where possible (for example, cell assays) unless indicated otherwise.

## Figures and Tables

**Table 1:** Summary of tumor allografts derived from Ras-tD chimera cells

*Stable cell line*	*Number of tumors/allografts*	*Average tumor mass (g)*
GFP	0/14	0
R-Ras	2/13	0.008±0.007
R-Ras-tH	6/12	0.019±0.012
H-Ras	18/18	2.09±0.51
H-Ras-tR	12/12	1.75±0.27

Mice were allografted with a bolus injection of cells stably expressing each Ras chimera into each flank, and the number of solid tumors formed per allograft is shown. Masses of resected tumors are shown±s.e.m.

**Table 2:** Summary of signaling, cellular and tumorigenic effects of Ras-tD chimeras

*Ras variant*	*PI3K*	*MAPK*	*Proliferation*	*Transformation*	*Tumorigenesis*
GFP	−	−	−	−	−
R-Ras	+	−	+	−	−
R-Ras-tH	+	+	+++	+ +	+
H-Ras	+++	+++	+++	+++	+++
H-Ras-tR	+++	+	+	+ +	+++
RNex-H-Ras	ND	ND	+++	+ +	+ +

Abbreviations: −, undetectable activity; +, weak, but detectable activity; +++, very strong activity; ND, not determined.
