# Supplementary figures and images for: Regulation of H-Ras-driven MAPK signaling, transformation and tumorigenesis, but not PI3K signaling and tumor progression, by plasma membrane microdomains
Source: Oncogenesis. 2016 May 30;5(5):e228–. doi: 10.1038/oncsis.2016.36 (PMC4945753; doi:10.1038/oncsis.2016.36)

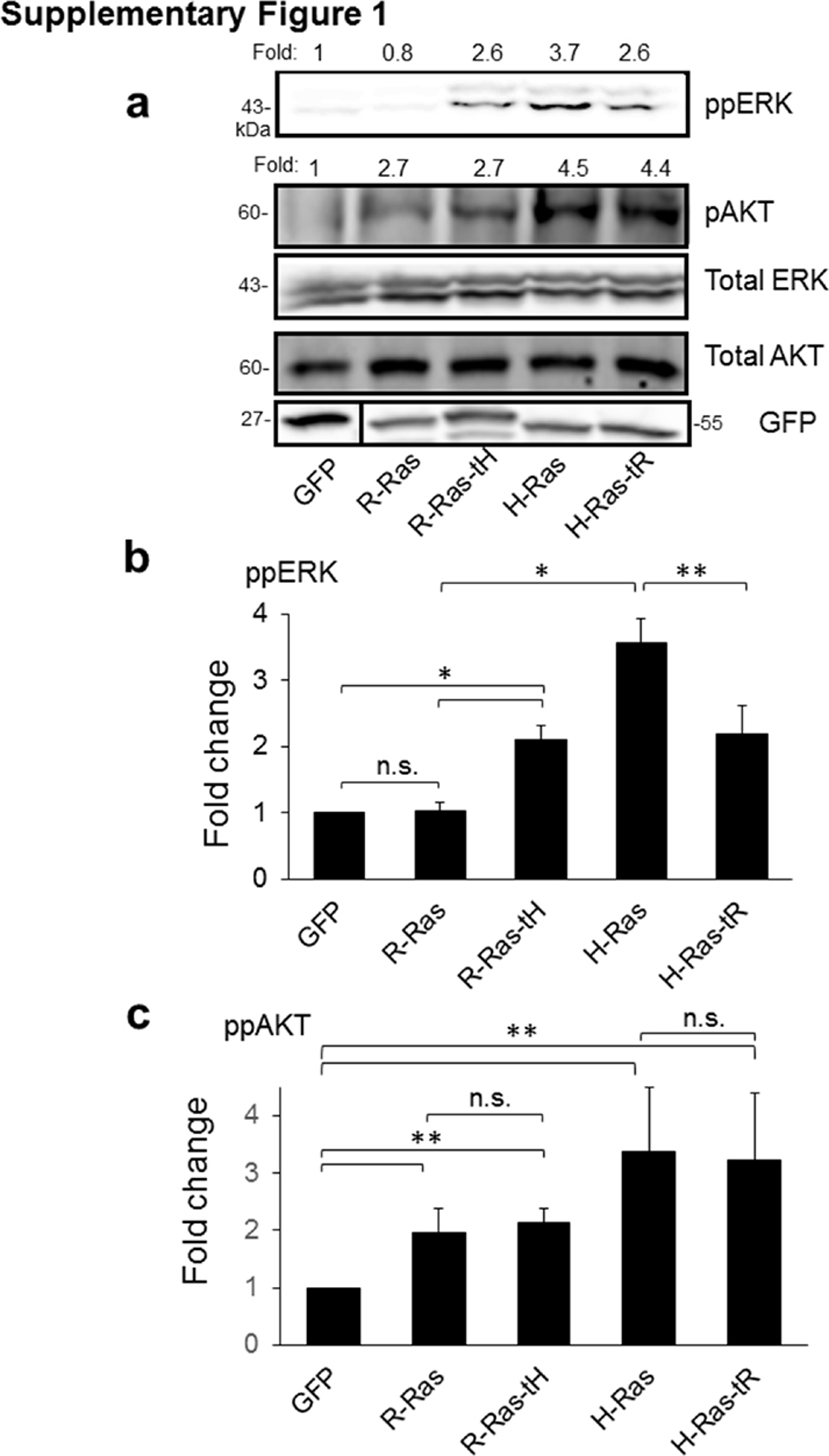

Supplement: Supplementary Figure 1 [file oncsis201636x1.tif]

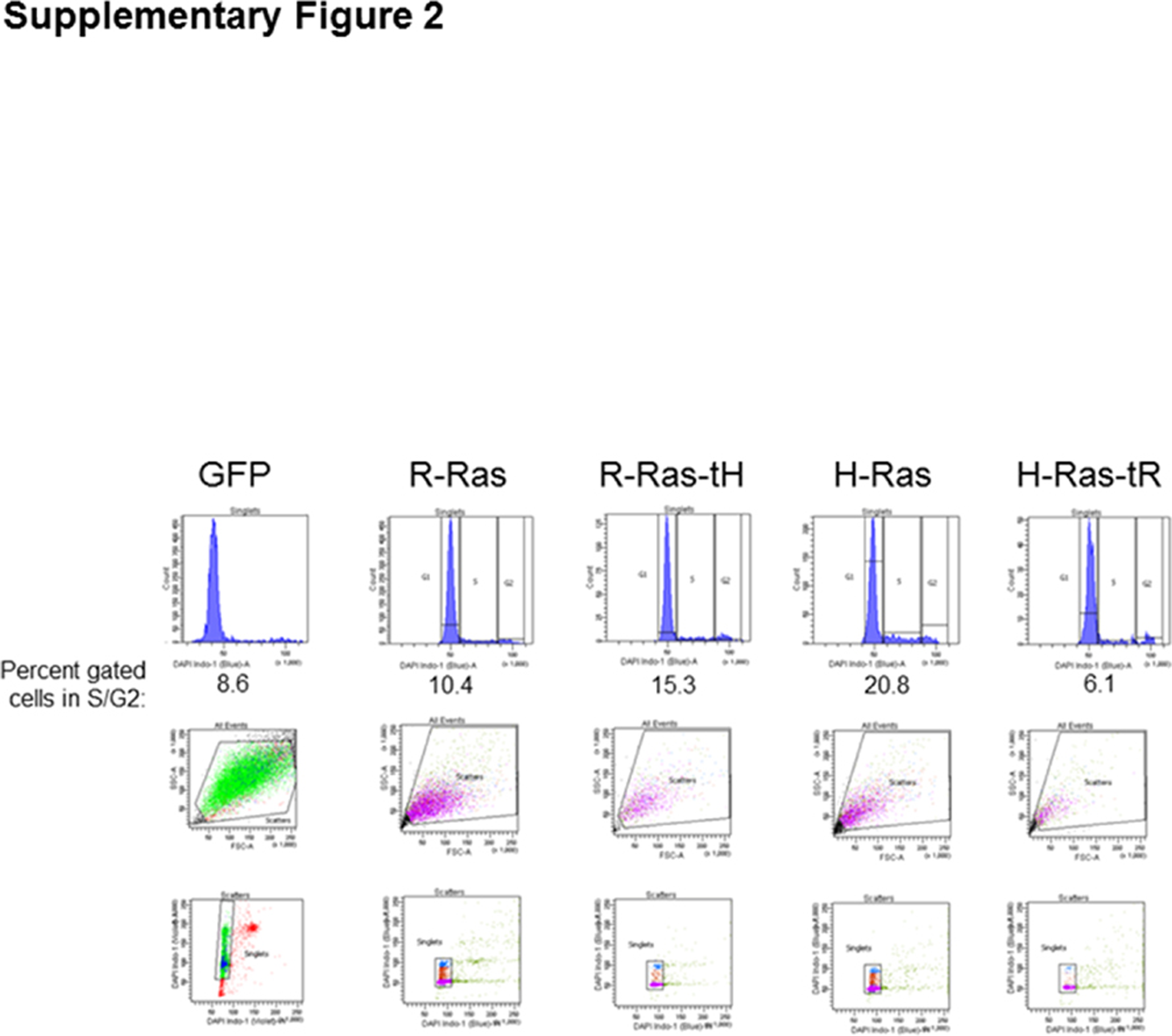

Supplement: Supplementary Figure 2 [file oncsis201636x2.tif]

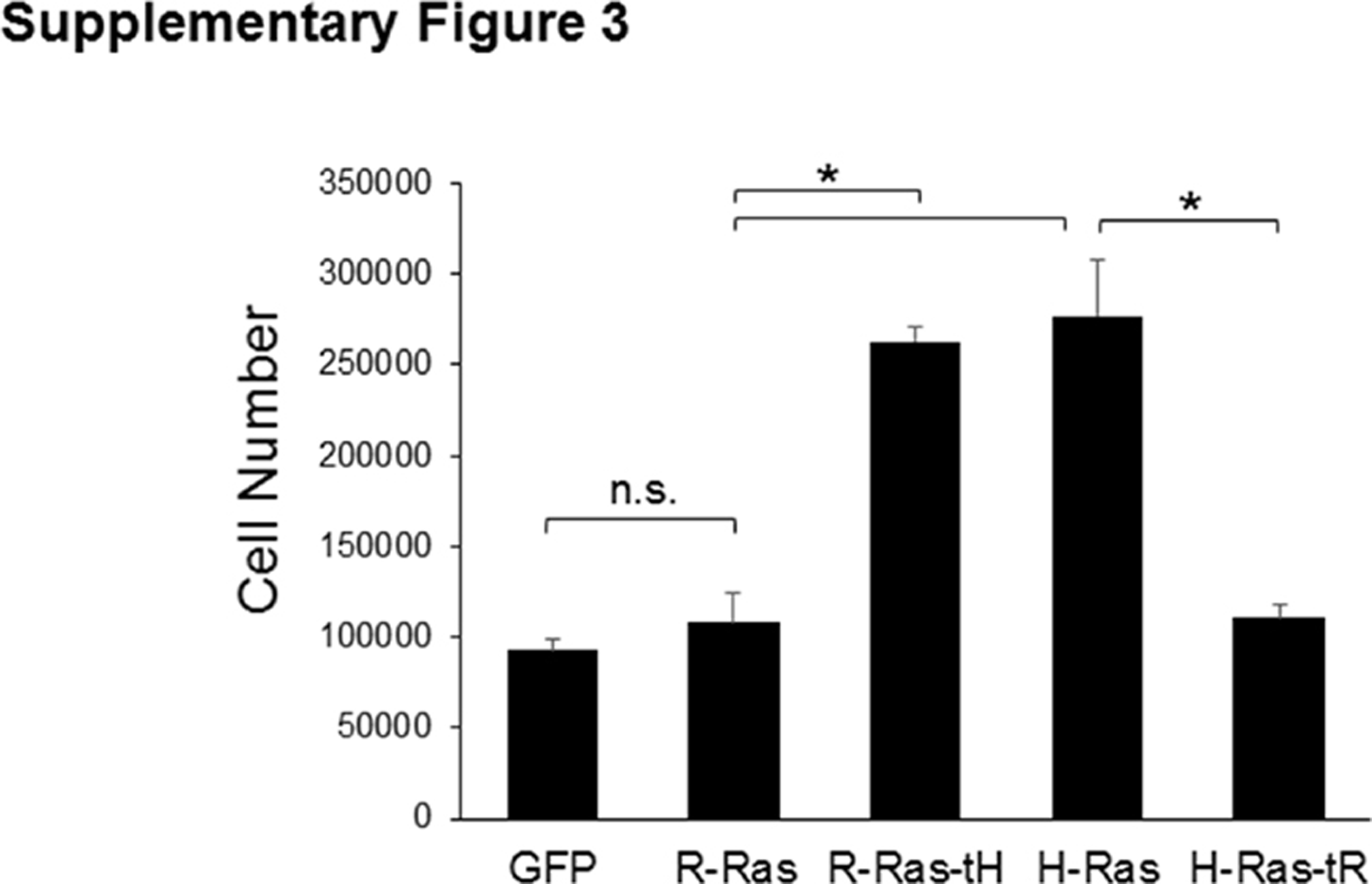

Supplement: Supplementary Figure 3 [file oncsis201636x3.tif]

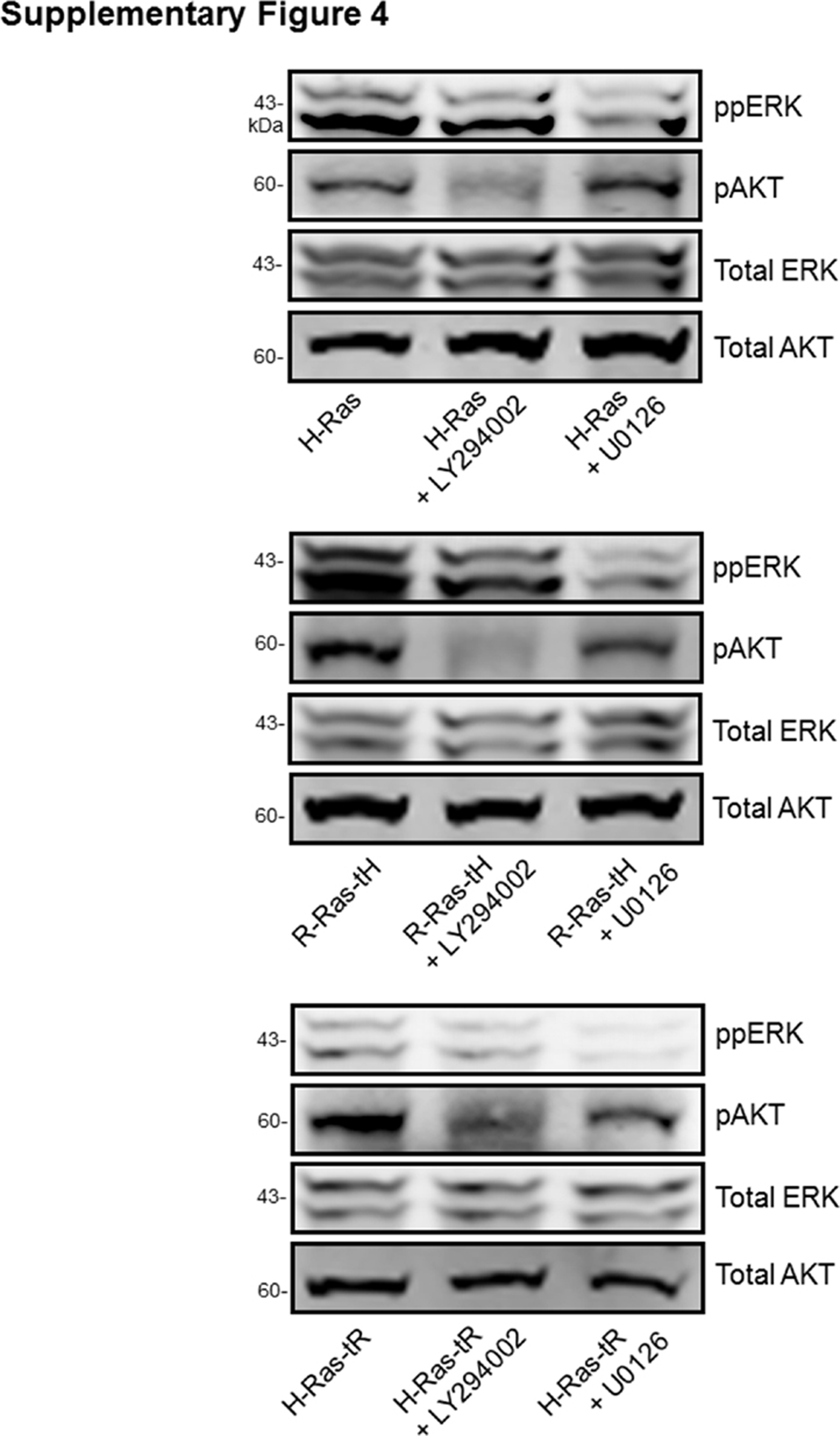

Supplement: Supplementary Figure 4 [file oncsis201636x4.tif]
