# Supplementary material for: Regulation of H-Ras-driven MAPK signaling, transformation and tumorigenesis, but not PI3K signaling and tumor progression, by plasma membrane microdomains
Source: Oncogenesis. 2016 May 30;5(5):e228–. doi: 10.1038/oncsis.2016.36 (PMC4945753; doi:10.1038/oncsis.2016.36)
Supplement: Supplementary Figure Legends [file oncsis201636x5.docx]

**Supplementary Figure 1. Ras targeting domains dictate MAPK signaling in HEK293 cells; both R-Ras and H-Ras stimulate AKT in HEK293 cells.** (a) HEK293 cells were transiently transfected with GFP-tagged Ras variants as indicated, and assessed for ERK and AKT activation after 72 h culture in low serum conditions. Phospho:total ratios are shown above the respective blots as a ratio to GFP. Lysates were subject to immunoblotting with α-GFP antibodies to indicate GFP or GFP-Ras expression. Representative of four independent experiments. (b,c) Fold change in phospho-ERK:total ERK or phospho-AKT:total AKT ratios compared to GFP control + s.e.m. *, *p* < 0.01; **, *p* < 0.05.

**Supplementary Figure 2. Cell cycle analysis of Ras stable cells.** NIH3T3 cells stably expressing the GFP or indicated GFP-Ras variants were maintained in low serum growth conditions for 72 h, then labeled with DAPI nuclear stain, and DNA copy number was assessed by fluorescence-activated cell scanning (FACS). Populations were gated for live single cells as shown; forward scatter (FSC)/side scatter (SSC) central panels show live cell brackets, lower panels show single cell gates. Brackets indicate predicted cell cycle phases. The proportions of each cell population in S/G2 phases per total singlet counts are shown below each DAPI histogram. Representative of two independent experiments.

**Supplementary Figure 3. The H-Ras tD modulates Ras-mediated proliferation in HEK293 cells.** HEK293 cells were transiently transfected with the indicated Ras variants, serum-starved, and seeded in triplicate wells at 5x10^4^ cells/well in media with low serum. Cells were harvested and counted at 72 h. Cells were harvested and counted 5 hr after seeding for 0 hr time points. Representative of three independent experiments. *, *p* < 0.001.

**Supplementary Figure 4. PI3K and MAPK inhibitors in Ras cells.** NIH3T3 cells stably expressing the indicated Ras variants were maintained in low serum conditions for 72 h, and the indicated inhibitors were added at 48 h. LY294002, 20 μm; U0126, 30 μm. Cells were lysed and processed for immunoblotting with the indicated antibodies. Representative of two independent experiments.
